# Gallbladder empyema: An atypical manifestation of acute cholecystitis

**DOI:** 10.1016/j.ijscr.2023.108530

**Published:** 2023-07-20

**Authors:** Luisa Trujillo-Guerrero, Edgar Javier Aguirre-Salamanca, Camilo Ramírez-Giraldo

**Affiliations:** a General Surgeon - Hospital Universitario Mayor – Méderi, Bogotá, Colombia; b Universidad del Rosario, Bogotá, Colombia

**Keywords:** Empyema necesitatis, Gall bladder, Cholecystitis, Abdominal wall abscess

## Abstract

**Introduction and importance:**

Acute cholecystitis is responsible for 44 % of emergency admissions to the emergency services with multiple complications such as empyema a necesitatis (EN). EN has a close relation with cholecystitis when the perforation of the gallbladder (GB) can lead to the formation of a biliary fistula. Patients can be asymptomatic, with late consultations, thus being a diagnostic challenge. Different techniques are described for cholecystitis and secondary abscess, therefore, the choice of the appropriate procedure should be the best one to reduce the high associated morbidity.

**Case presentation:**

We present a case of an 89-year-old patient, admitted for a sensation of a mass in the right hypochondrium with abdominal pain. He was taken to the operating room, finding a vesicular plastron with piocholecyst and perforation into the abdominal wall with abscess and fasciitis. Subtotal cholecystectomy was performed laparoscopically and an open approach in the abdominal wall, drainage of the abscess and debridement, leaving a negative pressure system.

**Clinical discussion:**

EN affects elder patients with high rates of morbidity, also GB empyema, which is related with its perforation and posterior fistulization, its external spontaneous perforation is much less frequent. Fistulas originated from the biliary tract are well described in the literature, with low incidence. They are related with improved diagnostic investigations and earlier implemented treatment by antibiotics and surgery.

**Conclusion:**

Biliary EN represents a very unusual complication of acute cholecystitis, its atypical presentation represents a diagnostic challenge, with very few cases documented and high mortality rates. Its management represents a challenge for the general surgeon, finding different approaches and surgical behaviors to take.

## Highlights

• There are multiple complications associated with this entity, one of them, is empyema a necesitatis.
• Perforation is a complication of cholecystitis, it can be generated by carcinoma or spontaneous and can lead to fistulization.
• The management on the acute phase, consists of analgesics, antibiotics and common treatment for acute cholecystitis.
• There are several surgical options such as cholecystectomy, cholecystostomy or abdominal drainage.

## 1 Introduction

Acute cholecystitis is responsible for a large number of admissions to emergency services, reaching 44 % in the United States [1]. There are multiple complications associated with this entity, one of them, and barely described in the literature, is empyema a necesitatis (EN) [2]. This pathology was described for the first time in 1963 [2,3] conditioning, in relation to cholecystitis, perforation of the gallbladder that causes the formation of a biliary-fistula. It occurs in older adults with a 3:1 ratio between women and men respectively. Patients with empyema a necesitatis can be asymptomatic, with late admission to the emergency room (ER) while mildly symptomatic [4], being a diagnostic challenge [2]. Regarding its management, different techniques have been described for the management of cholecystitis and secondary abscess, therefore, the choice of the appropriate procedure should be the best one to reduce the high morbidity associated [2]. We report a case of an EN in an 89-year-old male with a painful mass in the right hypochondrium, diagnosis was made by CT scan of the abdomen and was taken to laparoscopic cholecystectomy and open drainage of the abdominal wall.

We present the case according to the SCARE guidelines [5].

## 2 Case presentation

**Fig. 1:**
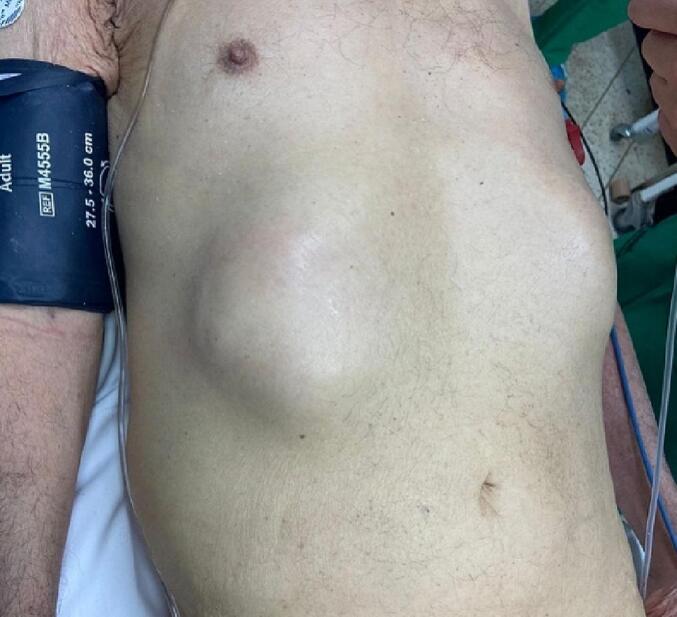
Upper right quadrant mass.

We present a case of an 89-year-old male patient with multiple comorbidities (congestive heart failure with a 48 % ejection fraction, ischemic cerebrovascular accident, atrial fibrillation on anticoagulant therapy CHA2DS2 VASC 7 points HAS BLED 3 points, chronic obstructive pulmonary disease, prediabetes, chronic kidney disease stage III), who was admitted due to an approximate two-month history sensation of a mass in right hypochondrium, associated with soft abdominal swelling, no redness or other skin changes. Clinical evaluation showed a tender palpable round mass in the upper right quadrant (Fig. 1), with no acute abdomen or other relevant findings. The patient had a stable clinical condition when admitted to the emergency department.

**Fig. 2:**
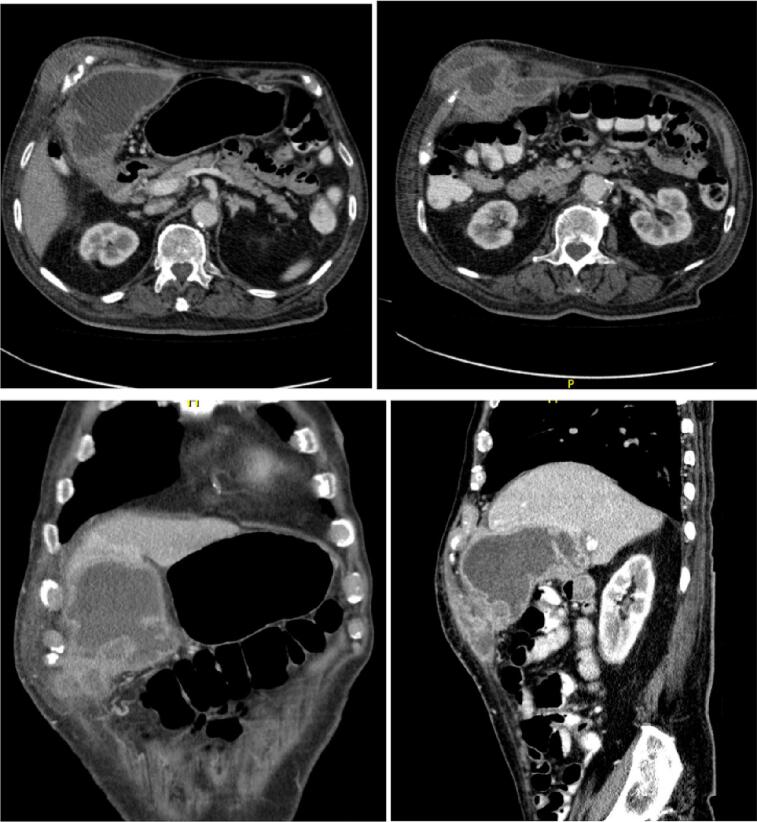
CT scan.

Within extension studies, a CT scan of the abdomen with contrast was performed, which showed an image compatible with acute perforated cholecystitis, with an associated multiseptated collection, with spontaneous drainage to the thoracoabdominal wall (Fig. 2).

**Fig. 3:**
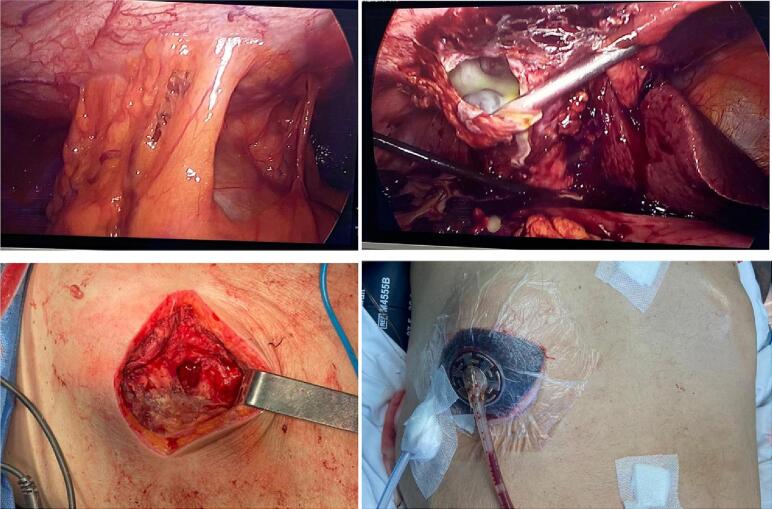
Intra-operative findings.

With these findings, he was taken to the operating room, finding a vesicular plastron with piocholecyst and perforation of the gall bladder into the abdominal wall, with abscess and fasciitis of the wall involving fascia and subcutaneous tissue. Subtotal cholecystectomy was performed laparoscopically and openly at the level of the abdominal wall, drainage of the abscess and debridement, leaving a negative pressure system on the abdominal wall (Fig. 3). Histological examination of the gallbladder showed changes of chronic and acute cholecystitis.

In the immediate postoperative period, the patient presented a torpid evolution, including septic shock managed in the intensive care unit (ICU) that required invasive ventilatory support and dual vasopressor therapy, with concomitant refractory hypotension. He finally passed away 20 h postop in the intensive care unit.

## 3 Discussion

Empyema a necesitatis (EN) is a clinical entity barely described in the literature that affects elder patients with high morbidity rates that is tightly related with its perforation and posterior fistulization as a complication of gallbladder empyema [2]. Gallbladder perforation (GP) is a complication of cholecystitis, and it can be caused by trauma, iatrogenic, carcinoma or spontaneous etiologies [6,7]. GP with subsequent fistula formation has been described in the literature, with perforation of the abdominal cavity or other structures such as the duodenum or liver being common; however, external spontaneous perforation is not as frequent, with very few cases in the literature; the first one having been described by Thilesus in 1670 [7]. Murad Aljiffry et al., described 3 categories of perforation, type I with free perforation into the peritoneal cavity, type II with localized perforation and type III with cholecysto-enteric fistulas [8].

Fistulas originated from the biliary tract are well described in the literature, with low incidence nowadays related to improved diagnostic research and earlier implementation of antibiotic and surgical treatments [9]. They can be both internal or external, most of these fistulae are internal with 77 % connected to duodenum, 15 % to colon and 10 % to stomach, jejunum, or other organs [10,11], nonetheless are spontaneous external biliary fistulae, they start in the biliary tract, and arise in multiple sites in the abdomen, before they rupture through the epidermis, as in this case, they are called empyema a necesitatis [12]. Cholecysto-cutaneous fistula usually open to the right hypochondrium, but other places have been described such as umbilicus (through falciform ligament), right iliac fossa, left hypochondrium and right gluteal region [10,[13], [14], [15]]. These entities mainly affect elderly patients and are generally associated with diffuse abdominal pain, which, when present, is very tolerable, and is associated with late consultation in the emergency department [4].

Having cholecystitis is a predisposing factor for peritoneal adhesions formation with the parietal peritoneum. With the concomitant inflammation and distension of the gall bladder, it adheres itself to the parietal peritoneum of the anterior abdominal wall [2,12]. This high abnormal distension leads to increased pressure in the gall bladder and cystic duct obstruction, generating decreased blood and lymph supply that may, in some cases, cause perforation, primarily of the fundus as blood flow is most distal in this is area, leading to peritonitis or an abscess surrounding it [2,11,14,16]. Usually, the perforation is contained by dense adhesions (created by the occurrence of previous episodes of inflammation) and commonly does not spread into the peritoneal cavity. It is also described as an acute-free perforation, subacute perforation, and chronic perforation which leads to peritonitis, abscess around the gallbladder and the formation of an internal or external biliary fistula respectively [11]. With the creation of the fistula, it burrows its way on the different planes over the abdominal wall to converge into one of the different sites described [12]. Nonetheless, in EN, a fistula is created between the perforation and the abdominal wall, exteriorizing towards the surface [2].

Clinical manifestations are highly variable, patients with empyema a necesitatis (wall abdomen abscess) can be asymptomatic, with late admission to the emergency room (ER) while mildly symptomatic [4], making it a diagnostic challenge [2,13]. These patients usually have a history of gallbladder stones or of untreated gallbladder disease [17]. Considering biliary fistulae, patients present abdominal pain with localized tenderness at the area of the fistula opening with systemic symptoms such as nausea, vomiting and fever [13]. Complications include sepsis (*Escherichia coli*, Coliforms and Klebsiella pneumoniae are the most common organisms found), malignant transformation of the fistulous tract and necrotizing soft tissue infections, as presented in our case, that might lead no necrotizing fasciitis [13,17].

Management of this condition is still controversial as it leads to more than 40 % risk of septic complications increasing mortality rates [18]. Management of the acute phase consists of analgesics, antibiotics and common treatment for acute cholecystitis and individualized surgical treatment. In some cases, a fistulogram could be performed to better identify the fistulous tract [12,13]. Surgical treatment described for these findings vary from cholecystectomy, cholecystostomy or abdominal drainage [2]. The preferred procedure is laparoscopic cholecystectomy, considering that complications might lead to subtotal cholecystectomy, conversion to open cholecystectomy or cholecystostomy due to alteration of the usual anatomy owing to the chronic inflammatory process [18]. Another alternative, if the patient does not tolerate general anesthesia is a two-stage approach, with an initial open cholecystostomy through a mini laparotomy or percutaneous technique, and a second interval cholecystectomy between 6 and 12 weeks after the first procedure [6,18]. Almayouf et al. J also consider it as the preferred option in patients deemed unfit to surgical observation of the patient allowing spontaneous closure of the fistulous tract [7].

The incidence of these pathologies has decreased due to the advent of diagnostic methods, the use of antibiotics and early surgical management of biliary tract disease [11,14].

Biliary empyema a necesitatis represents a very unusual complication of acute cholecystitis, its atypical presentation represents a current diagnostic challenge, with very few cases documented in the literature and high mortality rates. The management represents a challenge for the general surgeon, tasked with finding different approaches and surgical behaviors to consider establishing successful management. Considering what was previously described and the outcome of our patient, a more conservative management could be recommended in these patients, in whom the control and response to septic shock and surgical blow could lead to death. We decided on a more aggressive and less conservative treatment as the best option for the patient considering it provided a definitive treatment to the underlying etiology, and because he was on stable clinical condition during hospital admission. However, in the case of an octogenarian patient such as this one that is frail and who suffers from multiple comorbidities, a two-stage procedure treatment may have been the preferable option.

## Abbreviations

EN: empyema a necesitatis
GP: gallbladder perforation
ER: emergency room
ICU: intensive care unit

## Consent

Written informed consent was obtained from the family of the patient for publication of this case report and accompanying images. A copy of the written consent is available for review by the Editor-in-Chief of this journal on request.

## Ethical approval

Ethical approval for this study (Ethical Committee N° DVO005 2239-CV1681) was provided by the Ethical Committee of Universidad del Rosario, Bogotá, Colombia on 30 March 2023.

## Funding

This research did not receive any specific grant from funding agencies in the public, commercial, or not-for-profit sectors.

## Author contribution

Luisa Trujillo-Guerrero: Make substantial contributions to conception and design, acquisition of data, analysis and interpretation of data.

Edgar Javier Aguirre- Salamanca: Participate in drafting the article and revising it critically for important intellectual content.

Camilo Ramírez-Giraldo: Participate in drafting the article and revising it critically for important intellectual content.

## Guarantor

Camilo Ramírez-Giraldo.

## Research registration number

Not applicable.

## Declaration of competing interest

All authors declare no conflicts of interest.
